# Sex and Character

**Published:** 1908-03-14

**Authors:** 


### SEX AND CHARACTER.

It is unfortunate for the rest of humanity when a highly gifted author dies young, and in the case of Otto Weininger we have double cause for regret. After publishing the book, which bears the title of our article, he died by his own hand, somewhat over twenty-one years of age. We are therefore dealing with the thoughts of an eminently precocious brain, and naturally feel inclined to be sceptical of one who solved the problem of the "Sphinx without a riddle" almost before his beard was grown. Yet the leading ideas of the book hold our attention, particularly at a time when woman's agitation resounds throughout the land. Here, again, we might observe that the practical medical man should have more voice in the matter than many other classes of individuals who either are intoxicated with their own Byzantine rhetoric, or else have political axes to grind.

The "grund-idee" of Weininger's book on the matter of woman's demand for a vote is expressed in the following sentence in no ambiguous terms: "The real female element has neither the desire nor the capacity for emancipation in this sense (*i.e.* vote acquirement)." He then goes on to discuss the obvious fact that there are a considerable number apparently answering to the name "woman" who do require this vote. He suggests that these are cases where the female is more male in character and appearance, just as there are cases where the male is of entirely feminine aspect. Such people must have come under the observation of almost every medical man. Weininger then concludes that the vote-seeking woman is in reality abnormal, and in a small minority so far as the generality of the sex is concerned. He firmly persists in the view that to listen to the voice of sexual abnormalities is a mistake of the gravest nature. He points out that, broadly, history does repeat itself, and that there is a peculiar periodicity about the majority of remarkable movements. As early as the tenth century there was a woman's movement, and in the fifteenth and sixteenth there were writers like Sir Thomas Moore and Agrippa von Nettesheim, the former of whom declared that woman was the equal, the latter the superior, of man. If this periodicity could be established approximately as a scientific law we should be forced once more to consider what the causes are (always supposing that Weiningen's theory holds good) which tend to produce at given times a greater number whose behaviour is abnormal owing to abnormalities of sex.

We do not mean to assert, after the manner of Plato, that happiness is only possible where a philosopher governs the State, but we cannot help feeling that the scientific—nay, the economic and political—future of our country will largely depend on the efficiency of medical science. Theories fresh from the furnace can never be better tested than on the anvil of medical practice, where a man sees his fellow-men both at their best and their worst, where he can search for evidence to support or evidence to refute the most fascinating hypothesis. It is probable that such theories as those expressed by Weininger will not bear the test of rigid scrutiny, but praise is due to him even for attempting brilliantly to solve so vexed a question.

